# COVID-19-related adolescent mortality and morbidity in nineteen European countries

**DOI:** 10.1007/s00431-023-05068-z

**Published:** 2023-06-29

**Authors:** Jesus Cortés, Pedro Manuel Vargues Aguiar, Paulo Ferrinho

**Affiliations:** 1 NOVA National School of Public Health, Public Health Research Centre, Comprehensive Health Research Center, CHRC, NOVA University Lisbon, 1600-560 Lisbon, Portugal; 2 Global Health and Tropical Medicine, Instituto de Higiene e Medicina Tropical, Universidade Nova de Lisboa, Lisbon, Portugal

**Keywords:** COVID-19, Adolescents, Mortality, Vaccine, Environment

## Abstract

Prior to the COVID-19 pandemic, adolescents in most countries experienced a syndemic of malnutrition, obesity, deprivation, mental health problems, inequalities, and the effects of climate change. Today, other factors have added to this burden during the pandemic, and it is important to have an updated reflection. We aimed to assess the risk and protective factors for COVID-19-related adolescent mortality and morbidity in the European region. Three double models were fitted to analyze the relationship between different factors with the number of diagnosed cases and deaths. The 1a and 1b use a multiple Poisson regression. The 2a and 2b are optimized models that use the same variables as prior models but with backward selection with a *p* value < 0.05 as the limit. Finally, the 3a and 3b models (backward stepwise multivariable Poisson regression) include the variable “fully vaccinated.” All models used the at-risk population (15–19 years or total population) as a regression covariate (offset). Increased access to quality healthcare (IRR 0.68; CI 0.55–0.84), increased private sector involvement (IRR 0.86; CI 0.82–0.90), Gini coefficient (IRR 0.93; CI 0.88–0.99), and full vaccination (IRR 0.94; CI 0.90–0.99) represent protective factors of COVID-19 mortality in this population. Additionally, a positive association between pollution and mortality was found.

*  Conclusion:* Being fully vaccinated and having access to quality medical care are protective factors against COVID-19 mortality in this age group. Interestingly, the more the pollution, the greater the risk of dying from COVID-19. We stress the great importance of coordination between the public and private sectors to address crises such as the current one.

**Table:** 

**What is Known:**
• *Compared to other age groups, adolescents have been little studied, and most studies focused on mental health during the COVID-19 pandemic.*
**What is New:**
• *In this study, we show how in 19 European countries, different factors interact, such as socio-demographic, environmental, health system, and control measures with morbidity and mortality by COVID-19, in a very little studied age group as teenagers.*

## Introduction

The official end of the COVID-19 pandemic is considered near; however, the coronavirus continues to be present, in part related to the unequal vaccine coverage and the appearance of new variants [1].

Adolescence is one of the least studied population groups during the pandemic; it is a period of major bodily, psychological, and social development with significant health repercussions, which leaves teens included in the Global Strategy for Women’s, Children’s, and Adolescents’ Health and the Countdown to 2030 [2–4]. Although young people do not belong to the population group most affected by the pandemic, the negative effects on this age group of the containment measures taken and their possible medium- and long-term connections should be monitored [5] because children and youth are usually considered vulnerable populations and sensitive to sudden changes [6, 7].

However, even in the European context, where health and social assistance is greater than what is available in other regions, there are deficiencies, vulnerabilities, and inequalities between the continent’s countries regarding loss of family income, disruption of health systems, and the implementation of containment and control measures of the pandemic [7, 8]. As a result of these measures, young people had access to health care restricted and lost spaces of protection and interaction, such as schools or parks, which led to the detriment of their physical, mental, and socioemotional well-being [9]. Existing data do not point to a rise in youth mortality related to the COVID-19 pandemic, although there are suspicions of substantial indirect effects of the pandemic on their mortality [10].

There is a lack of reliable evidence on the topic, and the need for data on this specific population group is urgent. Circumstances such as forced confinement and the closure of schools that significantly affect the lives of young people should be monitored and planned considering different factors [11].

Having updated and disseminated statistics and indicators has been one of the major problems for many countries since 2020 [12], data that are essential for planning and making decisions at all levels, mainly in the age group of children and young people.

The study of the determinants and consequences of COVID-19 [13] is essential to guide the actions and government responses for social support. This study measures the determinants and predictors of COVID-19 mortality and morbidity in the population aged 15–19 years old, and focuses on containment measures, health system indicators, vaccination coverage, and environmental pollution as possible determinants and predictors of COVID-19 morbidity and mortality in the population aged 15 to 19 in the European region.

## Methods

This study uses data sources from 19 European countries to analyze the relationship between the main containment measures and characteristics of the countries with mortality and morbidity due to COVID-19 in the population aged 15 to 19 years.

The study design is a multiple-group exploratory study that uses aggregated data from countries in the European region.

### Variables and data sources

The variables included in the analysis consider the COVID-19 morbidity and mortality determinants, as reported in the literature [14], and were selected based on their plausibility with the outcomes studied; whereas in the literature, there is very little available information for this age group, also because these variables have been little explored in previous studies on mortality and morbidity in adolescents, and because these variables and indicators are essential factors of the countries likely to be improved.

The data sources were chosen from databases that hold up-to-date data on adolescents as reported by the official agencies of each country.

The two outcomes studied, the cumulative incidence of diagnosed COVID-19 cases and deaths, were extracted from the Global Health 50/50-COVID-19 project [15].

As covariates, we used the scores of the linking public health and security authorities, private health sector involvement in health response planning, access to quality healthcare, and public health vulnerabilities extracted from the Global Health Security Index (GHSI) 2021 [16], and in addition, the doctors per 100 k, hospital beds per 100 k, population housing per unit land area (km^2^), and the Gini coefficient (Table 1).

**Table 1:** Description of variables used in the present study

**Variables**	**Operationalization**
**Outcomes**	
** Cumulated diagnosed COVID-19 cases**	Absolute numbers, aged 15 to 19 years, reported until March 2022 by country
** Cumulated diagnosed COVID-19 deaths**	
**Covariates**	
** Linking public health and security authorities**	Scores from 0 to 100, with 100 representing the best performance for each country
** Private health sector involvement in health response planning**	
** Access to quality healthcare**	
** Public health vulnerabilities**	
** Doctors per 100 k**	
** Hospital beds per 100 k**	
** Population housing**	Per unit land area (km^2^) by country
** Gini coefficient**	0 to 1 (perfect equality) for each country
** Stringency index**	Rescaled from 0 to 100 (100 = strictest) for each country
** COVID-19 vaccines**	(Two doses) against COVID-19 (March 2022), all age groups per country
** Pollution PM2.5**	Air concentration toxic particles
** Log population**	Exposed risk population (adolescents 15 to 19 years, by country

Additionally, we used the covariate COVID-19 Containment and Health Index 2021 [17], which is based on thirteen policy response metrics (cancelation of public events, public gathering restrictions, public transport closures, general information campaigns, stay-at-home requirements, restrictions on internal movement, international controls, school recommendations, testing, contract tracing, face coverings, vaccination, and workplace closures) extracted from the Our World in Data organization [18]. We also extracted the percentage of persons (all ages) fully vaccinated (Table 1).

Finally, we also used the covariable concentration of contamination PM2.5, extracted by country (particles less than 2.5 μm in diameter; emitted by vehicles, industry, power generation, and domestic heating). Data from 2021 were obtained from the World Air Quality Report (Table 1) [19].

### Statistical analysis

In total, three double models were fitted (models 1a, 1b; 2a, 2b; 3a, 3b); the first three use the number of diagnosed cases per 15- to 19-year-old population per country as the outcome, and the subsequent three use the number of deaths per 15- to 19-year-old population per country with the same analytical approach (multiple Poisson regression [20, 21].

Models 1a and 1b use the following predictor variables: linked public health/security, private sector involvement, access to quality healthcare, public health vulnerabilities, containment, and health index, doctors per 100 k 15–19-year-old population, hospital beds per 100 k 15–19-year-old population, population housing per unit land area (km^2^), and the Gini coefficient.

Models 2a and 2b are optimized models that use the same variables as the prior models (but now using the backward stepwise multivariable Poisson regression, having a *p* value < 0.05 as the cutoff); the logarithm of the risk population was used as an offset variable in the Poisson regression model with the log link function.

Finally, models 3a and 3b (also used a backward stepwise multivariable Poisson regression) included the variable “fully vaccinated.” In this model, the variable stringency index was not included (to avoid collinearity since the containment and health index contains vaccine and vaccine policy indicators).

These models produce incidence rate ratios (IRRs) for SARS-CoV-2 infections and mortality due to COVID-19. Poisson regression was used with the offset variable computed by the exposed risk population, allowing to achieve incidence rate ratios based on incidences observed in country-people. All models used the population at risk (15–19 years or total population) as a regression covariate (offset [21]).

Analyses were performed using Stata software (StataCorp. 2017. Stata Statistical Software: Release 15.1. College Station, TX, USA: StataCorp LLC).

## Results

Nineteen countries of the European region that reported updated data for this age group were studied. A total of 1,388,164 adolescents were reported as confirmed cases by COVID-19 and included in this study. Table 2 shows some characteristics and indicators of the countries with variability in doctors per 100 k (Lithuania 75.4 points and Albania 14.3) and the same dynamic in public health vulnerabilities (Norway 93.6 and Albania 46.5).

**Table 2:** Characteristics of the countries studied (*N* = 19). ISO (International Organization for Standardization), doctors/100 k, public health vulnerabilities, and access to quality health care score

Country	ISO	Doctors/100 k^a^	Public health vulnerabilities^b^	Access to quality healthcare^c^
Albania	ALB	14.3	46.5	52.1
Croatia	HRV	35.5	58.1	57.1
Cyprus	CYP	23	52.7	66.5
Czech Republic	CZE	48.8	75.9	60.3
Estonia	EST	53.2	66.8	61.6
Finland	FIN	45.2	75.8	68.1
France	FRA	38.7	76.4	64.6
Hungary	HUN	40.4	64	56.3
Iceland	ISL	48.3	80.8	75.9
Letonia	LVA	37.8	54.7	53.6
Lithuania	LTU	75.4	57.4	57.9
Luxembourg	LUX	35.6	90.1	72.7
Malta	MLT	33.8	76.9	65.2
Montenegro	MNE	32.6	50.2	60.7
Netherlands	NLD	42.7	76.5	69
Norway	NOR	34.5	93.6	74.8
Portugal	PRT	60.8	68.6	65.2
Slovenia	SVN	36.5	64.4	68.4
Switzerland	CHE	50.9	78.6	71.1

^a, b, c^Source: Global Health Security Index 2019 [22]

**Fig. 1:**
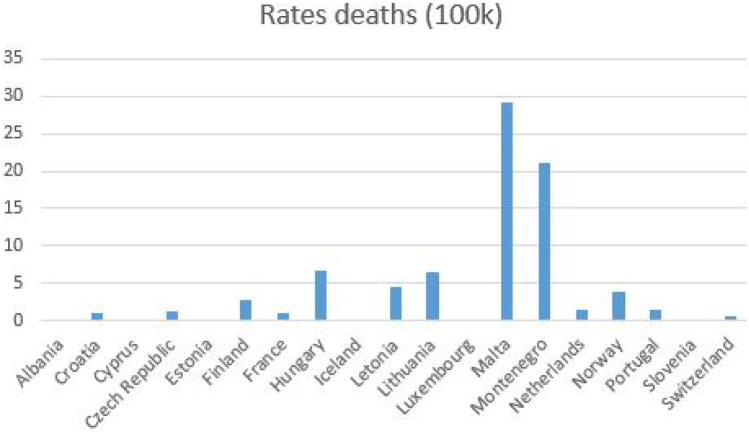
COVID-19 mortality in nineteen European countries as of March 2022, group (15 to 19 years old)

The mortality rate of 100,000 15–19-year-old population from COVID-19 is distributed variably; countries such as Malta and Montenegro show the highest mortality, but in countries such as Iceland and Slovenia, the mortality for this age group was very low (Fig. 1).

Regarding the diagnosed cases (Table 3), a greater link between public health and security (IRR 0.99; CI = 0.99–0.99), greater participation of the private sector (IRR 0.99; CI = 0.98–0.99), greater containment measures (IRR 0.93; CI = 0.93–0.94), fewer public health vulnerabilities (IRR 0.86; CI = 0.86–0.86), and full vaccination (IRR 0.82; CI = 0.82–0.82) were protective factors against becoming infected with SARS-CoV-2. The three models show that the higher the pollution, the higher the transmission of the virus and, therefore, more cases.

**Table 3:** Multiple Poisson regression of performance indicators of 19 European countries and diagnosed cases of COVID-19 in the population aged 15 to 19 years (March 2022)

	Model 1a				Model 2a				Model 3a			
Diagnosed cases	IRR	*p* value	CI 95%		IRR	*p* value	CI 95%		IRR	*p* value	CI 95%	
			LL	UL			LL	UL			LL	UL
Housing density	1.002	0.001*	1.002	1.002	1.003	0.001*	1.002	1.003	1.003	0.001*	1.003	1.003
Air pollution	1.097	0.001*	1.096	1.098	1.097	0.002*	1.096	1.098	1.023	0.001*	0.923	0.924
Hospital beds per 100 k	1.068	0.001*	1.068	1.069	1.070	0.001*	1.068	1.070	1.277	0.001*	1.276	1.278
Gini coefficient	1.038	0.001*	1.038	1.038	1.038	0.001*	1.038	1.038	0.971	0.001*	0.971	0.972
Doctors per 100 k	1.045	0.002*	1.045	1.045	1.045	0.001*	1.045	1.045	1.117	0.001*	1.117	1.117
Stringency index	0.914	0.001*	0.914	0.914	0.914	0.001*	0.914	0.914				
Linkage of public Health and security authorities	0.996	0.001*	0.996	0.996	0.997	0.001*	0.996	0.997	0.961	0.001*	0.961	0.961
Private sector involvement	0.982	0.001*	0.982	0.982	0.990	0.001*	0.982	0.992	0.973	0.001*	0.973	0.973
Access quality healthcare	1.588	0.001*	1.587	1.590	1.589	0.001*	1.587	1.590	1.722	0.001*	1.721	1.724
Public health vulnerabilities	0.861	0.001*	0.861	0.861	0.860	0.001*	0.860	0.861	0.982	0.001*	0.982	0.983
Vaccine COVID									0.823	0.001*	0.823	0.824
Log population	1(offset)											

*LL* lower limit, *UL* upper limit

*p value < 0.01

Model 1a—Number of obs = 18 − Model Prob > chi2 = 0.01

Model 2a—Number of obs = 18 − Model Prob > chi2 = 0.01; stepwise

Model 3a—Number of obs = 19 − Model Prob > chi2 = 0.01; stepwise + vaccines var

Regarding deaths (Table 4), the same variables of the previous model were considered. We highlight that for model 1, higher access to quality healthcare (IRR 0.68; CI 0.55–0.84), hospital beds per 100 k population (IRR 0.80; CI 0.72–0.88), greater participation of the private sector (IRR 0.97; CI 0.96–0.99), greater containment measures (IRR 0.86; CI 0.82–0.90), Gini (IRR 0.93; 0.88–0.99), and full vaccination (IRR 0.94; CI 0.90–0.99) were protective factors against mortality from COVID-19. It is important to mention that the variable “pollution” was shown to be a risk factor (IRR 1.13; CI 0.97–1.32), suggesting that the higher the pollution, the greater the risk of dying from COVID-19 after adjusting for the other covariables.

**Table 4:** Multiple Poisson regression of performance indicators of 19 European countries and confirmed deaths by COVID-19 in the population aged 15 to 19 years (March 2022)

	Model 1b				Model 2b				Model 3b			
Deaths	IRR	*p* value	CI 95%		IRR	*p* value	CI 95%		IRR	*p* value	CI 95%	
			LL	UL			LL	UL			LL	UL
Housing density	1.003	0.001*	1.002	1.004	1.003	0.001*	1.002	1.004	1.002	0.007**	1.001	1.003
Pollution	1.136	0.105	0.974	1.325								
Hospital beds per 100 k	0.804	0.001*	0.728	0.888	0.897	0.001*	0.850	0.947	0.924	0.017**	0.866	0.986
Gini coefficient	0.939	0.027**	0.889	0.993					0.923	0.008**	0.872	0.979
Doctors per 100 k	0.997	0.849	0.967	1.028								
Stringency index	0.861	0.001*	0.821	0.903	0.871	0.001*	0.835	0.908				
Linking public health/security authorities	1.028	0.001*	1.015	1.042	1.024	0.001*	1.012	1.037				
Private sector involvement	0.979	0.006**	0.964	0.994	0.973	0.001*	0.960	0.987				
Access to quality healthcare	0.758	0.05**	0.576	0.999					0.688	0.001*	0.557	0.849
Public health vulnerabilities	1.150	0.039**	1.007	1.312					1.170	0.008**	1.042	1.314
Vaccine COVID									0.945	0.017**	0.902	0.990
Log population	1(offset)											

*LL* lower limit, *UL* upper limit

**p value < 0.05, *p value < 0.01

Model 1b—Number of obs = 18 − Model Prob > chi2 = 0.01

Model 2b—Number of obs = 18 − Model Prob > chi2 = 0.01; Variables Gini, access to quality healthcare, doctors per 100 k, public health vulnerabilities, and pollution were analyzed in the model and excluded (*p* > 0.05); stepwise

Model 3b—Number of obs = 19 − Model Prob > chi2 = 0.01; + vaccines var. Pollution, doctors per 100 k, stringency index, linking public health/sec, and private sector involvement were analyzed in the model and excluded (*p* > 0.05); stepwise

## Discussion

Before the pandemic began, there was already evidence that adolescents suffered from a mixture of malnutrition, obesity, deprivation, mental health problems, inequalities, and climate change [23–25]; the scenario now seems worse because of the impact of the pandemic [26]; the results presented give some indication of the factors that contributed to that increased burden. There is little evidence of factors associated with adolescent mortality due to COVID-19. A study in the USA attributed deaths in children under 21 years of age to COVID-19, commonly related to obesity, asthma, and developmental problems [27].

This study addressed other causes that could be related to morbidity and mortality by COVID in a poorly studied age group (15 to 19 years). The Sustainable Development Goals (SDGs) focus on not leaving anyone behind and protecting children and young people [28, 29].

In this sense, the factors studied here are in agreement with other studies since the pandemic has shown that socioeconomic inequities, together with clinical aspects specific to individuals, play a determining role in mortality [30, 31]; this is an important dynamic to study and understand because it will allow us to mitigate the impact experienced by young people and provide key knowledge for future planning.

Our main results show that factors such as more hospital beds, Gini coefficient, number of physicians, access to quality healthcare, and linking between public health/security and public health vulnerabilities positively correlate with higher mortality and morbidity from COVID-19. These results might seem contradictory to what is expected, but it is not necessarily so; a study in the UK highlights that although at the beginning of the pandemic, young people showed good intentions to follow the rules and recommendations, in practice, this was not necessarily the truth; many young people are more likely to break the rules of social distancing [32]. In addition, the positive relationship between hospital beds/100 k and diagnosed cases of COVID-19 can be explained by the fact that more hospital beds imply greater resources, physical capacity, and possibilities to care for patients, leading to greater access and lower mortality, as our results show.

This study also showed that European countries present important differences in public health vulnerabilities. The multiple regression analysis of the diagnosed cases allowed us to identify some aggravating factors for the transmission and death by COVID-19 and some protective factors. Being fully vaccinated and having access to quality healthcare are two fundamental protective factors against mortality due to COVID-19 in this age group.

Environmental factors such as air pollution and its role in COVID-19 morbidity and mortality have been of study and concern [33]; in some studies, air quality alone is not a decisive protective factor in preventing the spread of the virus [34]. This must be immersed in a set of other factors. Therefore, we have included it in our model.

Of interest is that the higher the pollution, the higher the risk of dying by COVID-19; in this sense, reducing pollutants needs to be an important part of preventing severe cases of disease that lead to death. It is documented that pollution during the COVID-19 pandemic decreased in many parts of the world due to containment measures, limited mobility, and less use of cars, suggesting that there had not been this dichotomy, and the number of deaths could be even higher. This study found a positive association between mortality and environmental pollution, corroborated by other studies [35]. Urban growth, environmental controls, and how the built environment contributes to mortality and morbidity should be the subject of deep analysis at the local level of each country and subregion.

In this study, the stringency index variable was considered, which includes vaccines, vaccine policy, and school closures during the pandemic; the closure of schools covered primary, secondary, and university education, which means that our age group of study has been affected. Since the start of the pandemic, it was said that schools could be places of infections and that students could then transmit the virus to others at home, which generated a severe risk, especially for older adults; however, several recent studies report that this is not necessarily true, because youth-to-adult transmission is less than adult-to-youth transmission [36–47].

Some studies have reported that school restrictions do more harm than good and that they were executed in an empirical context [48–51].

It is important to say that, on the one hand, the restriction measures protect against morbidity and mortality from COVID-19. Nevertheless, on the other hand, they harm other areas of young people’s lives, as emotional abuse was likely exacerbated during this time of economic uncertainty and stress, adding to the increase in domestic violence [52]. Regarding the indicator used as a stringency index, which is a score on a set of restraint measures employed by countries, it presents differences between countries; Montenegro, Slovenia, and Luxembourg are among the countries with the highest scores, and France, Lithuania, and Hungary are among the lowest; however, it is necessary to mention that there is no linear relationship between this indicator alone and lower morbidity or mortality, which depends on a set of factors that interact dynamically.

We highlight the importance of coordination between the public and private sectors to address crisis such as the current one. This suggests that in many countries where health systems are shared, it is always important to work continuously with private organizations to increase the protection of the population.

As for another important aspect when dealing with young people in the European continent, where many countries possess a good level of development, better health systems, and better socioeconomic conditions than in other regions of the world, many of them trust themselves and enter into a so-called relaxing behavior, which meant an increase in cases, reflected in higher hospital admissions, a greater use of beds, and the need for more doctors and health workers to care for them. This finding is important for identifying interventions focused on modifying behavior and health education better targeted for future and present health crises.

It is important to report some limitations; the main one was the lack of data from selected large European countries for this specific age group, a problem in all countries worldwide. Additionally, we cannot fully ensure a causal association between diagnosed cases and death due to COVID-19 and the predictors considered. However, the criteria used in our models allow us to affirm that the study is a responsible approximation of reality. Another limitation is that confirmed infection in teens is still not as common as in older age groups, although there could not be enough variability to detect these associations. Another possible limitation not only of this study but also of COVID-19 studies, in general, that use “cases” as a variable is that the number of diagnosed cases can be underestimated as it depends on the testing policies carried out and the resources available. However, diagnosed cases reported in official sources are vital to understanding the level of virus transmission.

This study also has strengths, such as the use of the containment and health index in the multiple regression models; despite being considered a guide to evaluating measures on the spread of COVID-19 cases per country, it considers the thirteen most important actions that governments have implemented, from mobility to internal and external restrictions, among others, already detailed in the “Methods” section. Another strength is the inclusion of environmental variables, vaccinations, health system characteristics, and socioeconomic indicators to make the analysis robust.

An important lesson from this study is the importance of free and up-to-date information on the pandemic; reliable data are essential to healthcare decisions. Many models at the beginning of the pandemic failed due to an inability to update information quickly; the digital progress of information systems is an area that must be strengthened. The pandemic may cause investment in this arena to increase considerably [53].

Within the objectives of sustainable health and well-being development, we point to several protective factors for this population group in this study. Many countries were slowly moving toward development goals, and the pandemic affected that progress even more; in the case of European countries, there are still actions to be done and programs to be strengthened, and we hope this study will contribute to the laying of essential factors to be included in the current and future strategies of protection and attention.

The well-being of adolescents is called a priority. This age period represents a unique opportunity to acquire and develop human and social skills for later life [54]. The syndemic [16] has worsened social inequities, mainly in the least protected people. In many countries and contexts, the effect has been devastating, affecting all spheres of life of young people and adults, alternating between other vital aspects such as physical activity and healthy food consumption [55, 23].

Investing in interventions to favor the development and protection of European adolescents is a key part of meeting sustainable development goals and producing human capital for the future; it is estimated that for every dollar invested in adolescent health interventions, the health, social, and economic returns will be ten times greater [56].

Finally, and in line with the messages of different organizations, many mistakes were made during the pandemic despite prior preparation and knowledge. We believe that with this study, we have contributed by studying a diverse array of variables that are part of the well-being of young people. The indicators and the environments of the countries where people live are closely linked to health outcomes [57]; this study sought to study aspects related to COVID-19 cases and deaths. We believe that the risk factors identified in this study will undoubtedly serve to focus strategies and interventions addressing the pandemic’s almost irreversible current and future damage to the young population [55].

## Data Availability

Data are available upon reasonable request.
